# The Effect of Dark Chocolate Consumption on Arterial Function in Endurance Male Runners: Prospective Cohort Study

**DOI:** 10.3390/sports12120344

**Published:** 2024-12-13

**Authors:** Zacharias Vordos, Ifigeneia Deli, Maria Anifanti, Stefan Kluzek, Nikolaos Koutlianos, Evangelia Kouidi, Asterios Deligiannis

**Affiliations:** 1 Laboratory of Sports Medicine, Department of Physical Education and Sports Science, Aristotle University of Thessaloniki, 57001 Thermi, Greece; zvordos@hotmail.com (Z.V.); ifigeneia.deli@gmail.com (I.D.); manyfant@phed.auth.gr (M.A.); koutlian@phed.auth.gr (N.K.); kouidi@phed.auth.gr (E.K.); 2 Sports & Exercise Medicine and Trauma & Orthopaedic Surgery, University of Nottingham, Nottingham NG7 2UH, UK; stefan.kluzek@ndorms.ox.ac.uk

**Keywords:** polyphenols, dark chocolate, vascular function, arterial stiffness, endurance runners

## Abstract

Foods rich in polyphenols have beneficial effects on health. This study aimed to examine the impact of dark chocolate on endurance runners’ arterial function. Forty-six male amateur runners, aged 25–55, participated. The initial assessments included clinical testing, arterial stiffness measurements, and a cardiopulmonary exercise test. The participants then consumed 50 g of dark chocolate (70% cocoa) daily for two weeks, maintaining their usual training routine. After this period, the baseline assessment was repeated. The results showed significant improvements. Pulse wave velocity decreased by 11.82% (*p* < 0.001), and augmentation index by 19.47% (*p* < 0.001). Systolic brachial blood pressure reduced by 2.12% (*p* < 0.05), diastolic by 2.79% (*p* < 0.05), and mean pressure by 2.41% (*p* < 0.05). Central arterial pressure also decreased, with systolic by 1.24% (*p* < 0.05), diastolic by 2.80% (*p* < 0.05), and mean pressure by 2.43% (*p* < 0.05). Resting heart rate increased by 4.57% (*p* < 0.05) and left ventricular ejection time decreased by 4.89% (*p* < 0.05), particularly in athletes over 40. Exercise time increased by 2.16% (*p* < 0.05), heart rate (max) by 1.15% (*p* < 0.05), VO_2_max by 2.31% (*p* < 0.05), and anaerobic threshold shifted by 6.91% (*p* < 0.001) in exercise time and 6.93% (*p* < 0.001) in VO_2_max. In conclusion, dark chocolate improves arterial function in endurance runners, enhancing vascular health.

## 1. Introduction

### 1.1. Physical Activity and Vascular Function

Physical activity has profound impacts on the vasculature in humans [1,2]. Aerobic function significantly reduces the aortic systolic pressure and augmentation index (AIx). It improves pulse wave velocity (PWV), cardiac output, and ejection fraction, while resistance training has a beneficial effect on the aortic systolic and diastolic pressure and combined exercise has a favorable impact on the PWV and cardiac output [3,4,5,6,7,8]. The nature of these changes in function and structure are dependent not only on the characteristics of the training load but may also be modulated by other factors such as the type of diet, substantial energy consumption, and the use of antioxidates [9].

### 1.2. Polyphenols and Nitric Oxide

Polyphenols are a category of phytochemical compounds primarily known for their antioxidant properties, and they are found in foods such as fruits, vegetables, and products derived from plant-based foods like dark chocolate, tea, apples, red wine, and coffee [10,11,12,13,14,15]. A notable characteristic of polyphenols is their ability to enhance the bioavailability and bioactivity of nitric oxide (NO), a vasodilator that improves endothelial function and reduces arterial stiffness, thus shaping overall vascular function [16,17,18]. Arterial stiffness is a significant risk factor resulting from functional and structural changes in the arteries. Reducing vascular elasticity increases the burden on the cardiovascular system, as the heart must work harder to compensate for the increased load on the myocardium [19]. The potential benefits of studying the enhancing effect of an ergogenic substance, such as dark chocolate, on the indices of arterial function in subjects with long-term aerobic exercise, such as endurance runners, are promising.

### 1.3. Nitrates and Dark Chocolate

There is evidence that an increased intake of foods and supplements rich in nitrates may enhance the action of NO, strengthen the immune system, and bring to light beneficial properties in athletes and in patients with atherosclerosis and arterial hypertension [20,21,22]. Clinical studies have shown that NO contributes to the dilation of blood vessels by regulating blood flow, reducing blood pressure, and maintaining vascular homeostasis by preventing platelet aggregation and leukocyte adhesion to the endothelium. At the same time, it can cause a reduction in oxygen cost during physical exercise [23,24,25].

Notable is the effects of the food above on endurance athletes’ performance such as middle and long-distance runners [26,27]. Endurance capacity is one of the key components of good physical fitness, directly related to the ability of the circulatory and respiratory systems to support prolonged physical activity such as running [28]. A characteristic food rich in nitrates that appears to have ergogenic effects is beetroot, especially beetroot juice, which is particularly popular in the running community. However, despite its benefits, beetroot and its derivatives raise concerns due to their taste, which is not particularly appealing to most consumers. The solution to this comes from some studies reporting similar benefits from the consumption of chocolate, particularly dark chocolate [29].

### 1.4. Background and Research Objective

Several studies have explored dark chocolate consumption in both healthy individuals and patients, with mixed results, particularly regarding its effects on vascular function and the cardiovascular system. The key findings of these studies include a reduction in blood pressure and arterial stiffness, decreased inflammation, improved endothelial function, and enhanced blood flow through vasodilation [30,31,32]. However, research on endurance athletes, such as long-distance runners, is limited. This is particularly important, as endurance performance heavily relies on optimal arterial function to sustain oxygen delivery to working muscles. This study aimed to investigate whether short-term dark chocolate consumption can improve arterial function in endurance runners, providing new insights into the relationship between diet, arterial health, and athletic performance in runners.

## 2. Materials and Methods

### 2.1. Participants

There was an open-call invitation in the media and running clubs from various regions across Greece to participate in the study. The inclusion criteria for the participants were the following: healthy, male runners, aged 25–55 years, being amateurs, neither participating professionally in corresponding championships nor making a living from running, volunteering, with a training frequency of at least three times per week. The age range was chosen because it represents the typical age group of amateur endurance runners, which was the target population of this study. The participants’ experience in endurance running was not a direct criterion for inclusion or exclusion but was assessed only for the interpretation of the results. This ensured that all the participants were active amateur runners with regular involvement in the sport, without relying on the exact duration of their experience in the activity. The exclusion criteria were allergies or intolerances, cardiovascular diseases, metabolic disorders, the use of medications, smoking, chronic illnesses, and a diet rich in flavanols because the consumption of additional foods that contain flavanols, such as extra chocolate, tea, and apples, may obscure or interact with the effects attributed to dark chocolate.

### 2.2. Study Design

**Figure 1:**
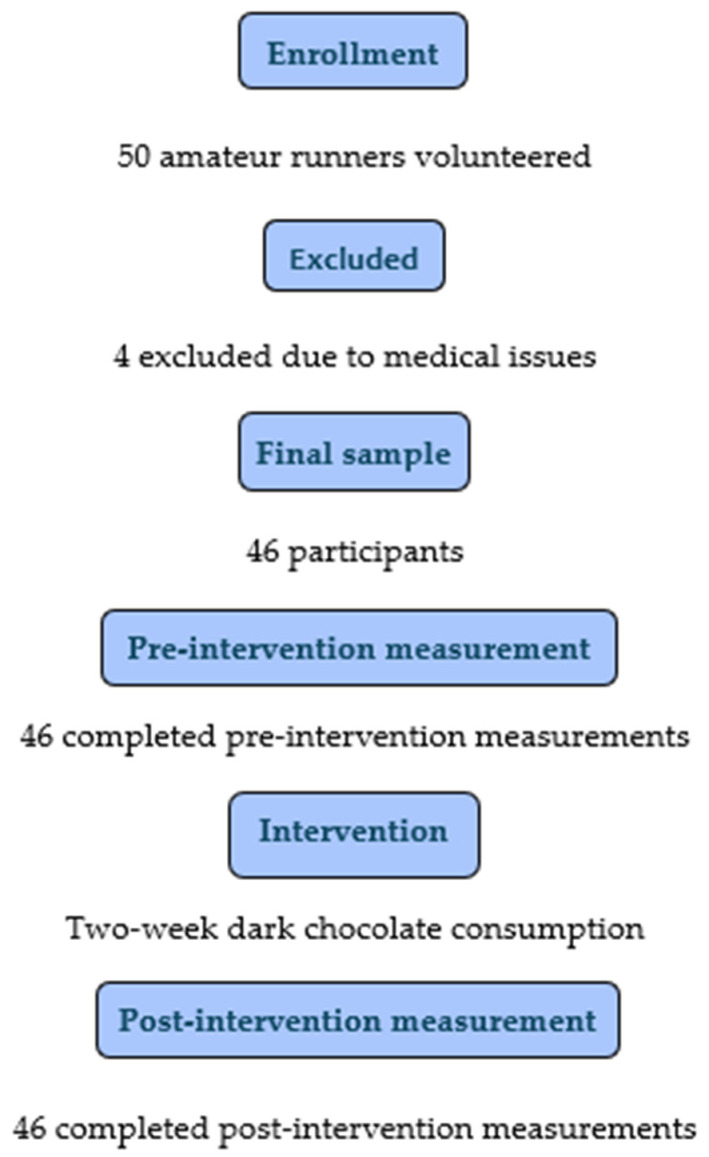
Study flow diagram.

All the participants were informed about the purpose and procedures of the study and completed a written consent form after being briefed on the ethical committee guidelines and the recommendations of Aristotle University of Thessaloniki. The study protocol was approved by the Ethics Committee of Aristotle University of Thessaloniki (Protocol number 161/2023). After providing signed consent to participate in the study, they completed their respective medical history forms. Following this, they underwent a clinical examination which included recording anthropometric characteristics, weight and height measurements, blood pressure measurement, and resting electrocardiogram. Athletes found to be clinically healthy were recruited and underwent an initial evaluation of arterial stiffness indices. The intervention consisted of 50 g of dark chocolate consumed daily for two weeks, as indicated by a recent systematic review [30]. After two weeks, all the baseline measurements were repeated (Figure 1). Post-intervention measurements were conducted on the day following the completion of the two-week dark chocolate intake period.

All the participants were provided with dietary and training guidelines. Specifically, they were required to abstain from alcohol and to avoid consuming any other supplements and ergogenic aids during the intervention period. They were advised not to take anti-inflammatory medications and should not regularly consume foods rich in nitrates (e.g., beetroot) and flavonoids (e.g., green tea, onions, apples, and wine). However, they could continue to eat other fruits and vegetables. Furthermore, they were instructed not to consume additional types of chocolate and to replace a daily snack with the designated chocolate to prevent excessive calorie intake, which could lead to weight gain. They were to maintain their normal diet otherwise [29]. Records of the foods and fluids consumed at least 24 h before each measurement were required. The chocolate should be consumed around 10:30–11:00 a.m. or two hours before training, and on measurement days, it should be taken 24 h before the assessment [33,34]. Additionally, it was consumed alone and not with other foods. The last intake of chocolate should occur 24 h before each measurement. Ideally, the chocolate should be stored in a cool, dry place (around 18 °C) and not in the refrigerator or a warm area where it could deteriorate. Throughout the experimental process, the participants were to follow a consistent training program, which should be recorded and communicated to the researcher for final evaluation.

### 2.3. Instruments and Measurement Procedures

On the day of the measurements, the athletes refrained from training and were instructed not to consume heavy meals or foods with vasodilatory properties at least three hours before each measurement. All the measurements were conducted in the morning, between 9:00 and 11:00. The measurements took place in an environment suitable for the test, ensuring adequate privacy and quietness. To ensure hemodynamic stability, the athletes remained supine, resting for about 10–15 min [35]. The same researchers conducted all the measurements. The arterial stiffness indices PWV and Aix, brachial and central blood pressure, pulse pressure (PP), middle arterial pressure (MAP), PP ratio, and left ventricular ejection time (LVET) assessment were performed non-invasively using the Complior Analyse from the French company Alam Medical, which has been validated for clinical use in previous studies [36,37]. Subsequently, brachial arterial pressure was recorded and entered into the Complior software v 1.9.4. (1.9.4.12) along with the anthropometric data (weight and height). Blood pressure was measured using the Omron M6 Comfort sphygmomanometer.

The right carotid and femoral arteries were measured [38]. Complior device sensors were placed at these two points. A minimum recording time of 10 cardiac cycles was used at each measurement point according to the 2024 recommendations for the validity of non-invasive device measurements, such as Complior [38]. The choice of measuring carotid–femoral pulse wave velocity (cf-PWV) was made because pressure waveforms can be quickly recorded at these sites, as the distance between the two points is large enough to allow for an accurate calculation of the time interval between the two waves that are simultaneously recorded on the Complior monitor. Additionally, it reflects the elasticity of the arterial wall related to the aorta [39].

Immediately after assessing the vascular parameters using Complior, the participants moved to the fatigue and cardiopulmonary exercise testing unit of the Sports Medicine laboratory, where they underwent a cardiopulmonary exercise test (CPET). During the graded cardiopulmonary exercise test, submaximal and maximal physiological variables were evaluated, allowing the assessment of cardiopulmonary capacity, exercise performance, and the comparison of values between one or more individuals [40]. The protocol used in this maximal test was the Bruce protocol, which is widely recognized and validated for assessing cardiovascular fitness in clinical populations [41].

To establish the minimum important difference (MID) for each variable, two different methods were combined as recommended by recent studies on clinical significance: the anchor method and the effect size method in order to determine whether the observed differences were above, below, or at the same level as the MID threshold [42]. The anchor method was used to link the self-reported assessments of the participants with the effect of dark chocolate consumption. Specifically, the participants were asked to answer the question, “How would you rate the effects of dark chocolate consumption?” which used a Likert scale to evaluate the perceived effect of dark chocolate consumption. Their responses were categorized on a five-point scale (very positive, positive, similar, negative, and very negative), allowing for the estimation of the percentage of participants who reported improvement. This method provides a subjective but valuable approach to assessing the clinical significance of the observed differences [42]. The combined use of the anchor method and the effect size, as described below, enhances the validity of the findings, confirming whether the statistically significant changes observed were also clinically significant for the athletes.

### 2.4. Dark Chocolate

A commercially available solid dark chocolate with a 70% cocoa content was used [30]. Commercial products were selected because they are certified, ensuring consistent manufacturing conditions and preventing ingredient and polyphenol content differences. The chocolate ingredients included cocoa mass; sugar; cocoa butter; emulsifier—soy lecithin; natural vanilla extract; and cocoa solids at 70%.

A 50 g serving of dark chocolate provides 1225 kJ (292.5 kcal) energy. It contains 20.5 g of fats, with 13.25 g (64.6%) being saturated fats. The carbohydrate content is 24.0 g, of which 14.0 g are sugars. Additionally, the serving provides 4.0 g of protein and 0.100 g of salt.

Table 1 presents the content of total polyphenols and catechins (catechin and epicatechin). The athletes consumed 50 g of dark chocolate (1222.5 mg total polyphenols and 577.5 mg catechins) daily for two weeks. The dose of 50 g of dark chocolate was selected based on previous studies that investigated the effects of dark chocolate on vascular function. This dosage has been shown to provide a sufficient amount of polyphenols, including catechins, to produce measurable effects without being excessive [30].

### 2.5. Statistical Analysis

For the estimation of the sample size, the G*Power software (v. 3.1.9.7) was used. The total sample size was calculated to ensure the accuracy of the estimates and the statistical power of the study. An effect size of Cohen’s d = 0.5 was applied for detecting statistical differences, with a statistical power (1-Type II error probability) set at 0.90 and a significance level (α) of 0.05.

Before applying the statistical tests, all the assumptions were checked, such as the normality test of the distribution of the variables. Due to the sample size, the Shapiro–Wilk normality test was applied. The parametric paired *t*-test was used for dependent samples to evaluate differences between the measurement stages. An effect size test using Cohen’s d index was applied to limit misestimation due to sample size and standard error and to confirm the exploration of the relationships between variables. For the correlation analysis, the Pearson correlation method was used. The *p*-values are presented in the result tables to evaluate statistical significance.

To evaluate the clinical significance of the results, the MID was calculated, which refers to the minimum difference considered clinically significant for the population of endurance runners. The calculation of MID was based on the effect size according to Cohen’s d. Initially, the standard deviation (SD) of the mean difference between measurements was calculated. Subsequently, for the calculation of MID, the SD was multiplied by values reflecting the effect size according to the thresholds defined by Cohen. Specifically, for small effects (Cohen’s d ≤ 0.399), the coefficient 0.2 was used; for moderate effects (Cohen’s d between 0.400 and 0.799), the coefficient 0.5 was used; and for large effects (Cohen’s d ≥ 0.800), the coefficient 0.8 was used [36]. This method allowed the clinical interpretation of the statistical results, ensuring that the observed differences were not only statistically significant but also clinically significant for the population of endurance runners. In this way, it was possible to assess whether the consumption of dark chocolate significantly affected the runners’ performance. Statistical analysis was conducted using IBM SPSS Statistics ver. 29, with statistical significance at *p* < 0.05.

## 3. Results

Fifty amateur male runners volunteered to participate in the study. Of them, four individuals were excluded due to medical issues. Thus, the final sample consisted of 46 healthy men, amateur endurance runners aged 40.69 ± 9.04 years. The athletes were divided into two subgroups: young runners (under 40 years) and middle-aged runners (over 40 years). Table 2 presents the clinical characteristics of the participants.

**Figure 2:**
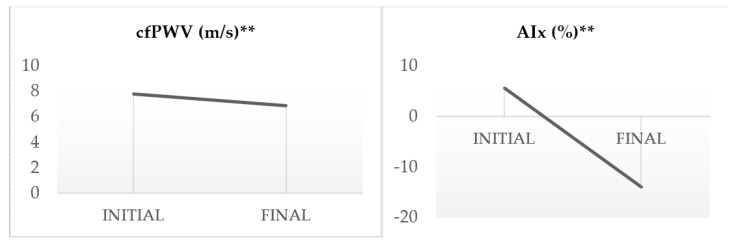
Variability of arterial stiffness indices cfPWV and AIx. Statistical significance was assessed using a *t*-test, **: *p* < 0.001.

**Figure 3:**
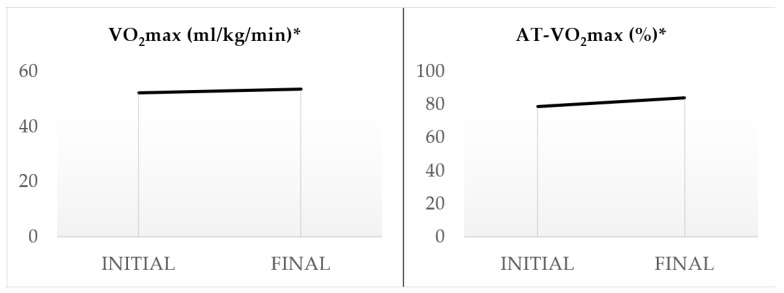
Variability of VO_2_max and AT-VO_2_max indices. Statistical significance was assessed using a *t*-test, *: *p* < 0.05.

**Figure 4:**
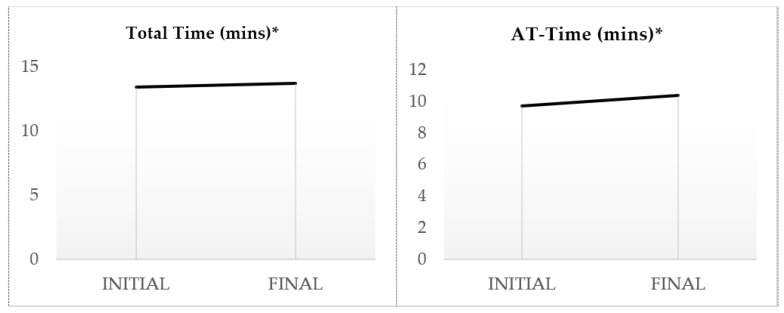
Variability of total exercise time and AT-Time indices. Statistical significance was assessed using a *t*-test, *: *p* < 0.05.

The consumption of dark chocolate reduced the most robust indices of arterial stiffness for all the participants (aged 25–55) (Table 3). Specifically, there was a significant reduction in cfPWV by 11.82% (*p* < 0.001) and AIx by 19.47% (*p* < 0.001) (Figure 2). Regarding brachial blood pressure, the systolic pressure (SBP) was significantly reduced by 2.12% (*p* < 0.05), diastolic pressure (DBP) by 2.79% (*p* < 0.05), and MAP by 2.41% (*p* < 0.05). Similarly, central arterial pressure showed a significant drop. SBP decreased by 1.24% (*p* < 0.05), DBP by 2.80% (*p* < 0.05), and central MAP by 2.43% (*p* < 0.05). Notably, there was also a significant increase in the resting heart rate (HR) by 4.57% (*p* < 0.05) and a decrease in the LVET by 4.89% (*p* < 0.001), especially in athletes over 40 years old. Furthermore, the exercise time in the CPET increased by 2.16% (*p* < 0.05), the HR (max) by 1.15% (*p* < 0.05), the VO_2_max by 2.31% (*p* < 0.05), ventilation (VE) by 3.77% (*p* < 0,05), and the anaerobic threshold shifted by 6.91% (*p* < 0.001) in exercise time and by 6.93% (*p* < 0.001) in the percentage of VO_2_max (Figure 3 and Figure 4).

Of the total participants, 23 were young adults under the age of 40, and 23 were middle-aged, over the age of 40. From the analysis conducted on those under 40, a significant reduction in cfPWV by 12.05% (*p* < 0.001) and AIx by 26.29% (*p* < 0.001) was observed, along with an increase in the total exercise time during the CPET by 2.23% (*p* < 0.05), a shift in the point of achieving AT with a time increase of 7.58% (*p* < 0.05), an increase in the percentage of VO_2_max at AT by 6.34% (*p* < 0.05), and an increase in pulmonary ventilation (VE) by 6.13% (*p* < 0.05) (Table 4).

In athletes over the age of 40, an increase in HR (rest) by 7.86% (*p* < 0.05) was observed, along with a reduction in brachial and central MAP by 2.49% and 2.50%, (*p* < 0.05), respectively, a decrease in cfPWV by 11.64% (*p* < 0.05), a reduction in AIx by 12.65% (*p* < 0.001), and a decrease in LVET by 6.40% (*p* < 0.05). In the CPET, there was an increase in the total exercise time and HR (max) by 2.08% and 1.32%, respectively (*p* < 0.05), along with an increase in the time to reach AT by 6.34% (*p* < 0.001) and in the percentage of VO_2_max at AT by 7.56% (*p* < 0.001) (Table 5).

The correlation analysis revealed a significant positive linear correlation between both the initial and final cfPWV and several key factors, as age (r = 0.392 and r = 0.397, *p* < 0.01), BMI (r = 0.329 and r = 0.331, *p* < 0.05), SBP (r = 0.362, *p* < 0.05 and r = 0.399, *p* < 0.01), DBP (r = 0.357 and r = 0.407, *p* < 0.05), and HR (r = 0.323 and r = 0.326, *p* < 0.05). Furthermore, a positive linear correlation was found between training experience, both in the initial and final measurements, and VO_2_max (r = 0.844 and r = 0.850, *p* < 0.01), total exercise time (r = 0.675, *p* < 0.01 and r = 0.694, *p* < 0.05), and time at AT (r = 0.519 and r = 0.514, *p* < 0.01). However, no corresponding correlations were observed with the differences between the two measurements.

The results from the calculation of the MID showed significant clinical improvement in most statistically significant parameters (Table 3, Table 4 and Table 5), which is also confirmed by the results of the anchor method. Specifically, 87% of the participants reported that they noticed an improvement in their performance, 10% stated that they remained at the same level, and a smaller percentage of 3% reported a decline in their performance (Table 6). 

## 4. Discussion

This study focused on the effect of dark chocolate on arterial function in male endurance runners, a topic of growing interest due to the cardiovascular benefits of the flavonoids in dark chocolate. Cardiovascular function, particularly arterial health, is crucial for endurance performance, as the ability to efficiently deliver oxygen to muscles directly affects endurance and overall performance. The relationship between dark chocolate and arterial function may offer valuable insights into how diet can enhance athletic performance and contribute to broader cardiovascular health. The results indicated a positive impact on arterial function, improving the primary indicators of arterial stiffness and significant cardiopulmonary parameters.

Regarding the initial measurement, the indicators of arterial stiffness were within normal levels, consistent with the study of Van de Laar et al. (2011), which showed that lifelong exercise, particularly systematic exercise, positively contributes to arterial elasticity [43]. This study concluded that even small increases in high-intensity systematic exercise from adolescence to adulthood can prevent arterial stiffness in youth. Thus, considering the years of training experience of the young adults in the present study, over 10 years, it is confirmed that aerobic exercise contributed to their physiologically low values of cfPWV and AIx. The individuals aged 40–55 years, despite their older age, had fewer years of training experience (less than ten years) and exhibited higher values in the indicators of arterial stiffness, both in cfPWV and AIx. These results are consistent with many studies reporting that the elasticity of blood vessels decreases as age increases [5,44,45]. The positive linear correlation between age and reduced elasticity, as measured by cfPWV, was also confirmed by the positive correlation found in the present study.

A highly positive linear correlation was also found between training experience and performance during CPET. Specifically, the results indicated that training experience plays a crucial role in shaping VO_2_max and the onset of AT. However, although there was a negative linear correlation between arterial function and cardiopulmonary capacity, this was not statistically significant. This finding contrasts with studies such as those by Fernberg et al. (2017), which showed a high correlation between cardiopulmonary capacity and arterial stiffness in young adults, emphasizing the importance of maintaining or increasing this capacity in conjunction with maintaining an ideal body mass index [46].

After dark chocolate consumption, a significant reduction was observed in the primary prognostic indicators cfPWV and AIx for all the participants. These results are consistent with the meta-analysis by Azad et al. (2021), which found that weekly cocoa product consumption significantly reduces PWV and AIx indicators in studies with intervention durations of less than four weeks, primarily involving healthy men [30]. Additionally, a decrease in both central and peripheral blood pressure was noted. The effect of dark chocolate on blood pressure regulation is also supported by the findings of Taubert et al. (2007), which showed that the consumption of polyphenol-rich dark chocolate as a part of a regular diet effectively reduces blood pressure and contributes to the formation of the vasodilator NO [47]. Improving endothelial function is also supported by the findings of Engler et al. (2004), who, however, did not observe improvements in blood pressure after dark chocolate consumption [33].

Furthermore, the results indicated a significant positive shift in the AT and a small but significant increase in VO_2_max (2.31%, *p* < 0.05). Notably, there was also an improvement in the total exercise time. These findings are consistent with those of Patel et al. (2015), whose study reported improvements in VO_2_max, AT, and performance after dark chocolate consumption for two weeks. However, in contrast to the present study, the study by Patel et al. (2015) was conducted on cyclists, showing a greater increase in VO_2_max (6%, *p* < 0.05) [29]. At this point, the study by Davison et al. (2011) is also relevant, as it found that the consumption of dark chocolate enhances antioxidant capacity before exercise and reduces oxidative stress. This improvement in antioxidant status may contribute to enhanced aerobic capacity and vascular function in endurance runners, supporting our findings regarding the effect of chocolate on VO_2_max and other performance parameters [48].

The effects observed in the study are primarily assigned to dark chocolate, which is rich in polyphenols. Specifically, the improvement in these indices is attributed to the increased bioavailability of NO, which reduces arterial stiffness and improves endothelial function. The latter is directly related to the plasma concentration of epicatechin [49]. Flavanols, such as epicatechin, reduce the synthesis of a potent vasoconstrictor produced by endothelial cells, endothelin-1, resulting in greater NO bioavailability due to the reduction in reactive oxygen species formed by NO and O2 interactions [50,51,52]. An imbalance between NO and endothelin-1, which are in opposition, is characteristic of endothelial dysfunction and is significant for the progression of vascular disease [53]. Acute epicatechin intake can cause vasodilation by releasing NO from endothelial cells within one hour of consumption, peaking about two hours after ingestion [54].

Moreover, due to their antioxidant properties, polyphenols reduce oxidative stress, favoring the reduction in arterial stiffness and enhancing endothelial function [17]. Thus, reducing oxidative stress helps maintain vessel elasticity, improving the PWV and AIx indices. The anti-inflammatory properties of polyphenols were also crucial in enhancing these indices by reducing chronic inflammation in the vessels, thereby improving vascular health through a significant reduction in the values of the predictive indices of arterial stiffness. This is confirmed by Fleenor and Berrones (2015), who highlighted the importance of inflammation in large arteries as a critical factor in vascular aging and arterial stiffness, attributing the reduction in PWV to increased NO bioavailability through elevated plasma epicatechin levels [19].

Alongside the reduction in blood pressure mentioned above, an increase in HR (rest) was observed, accompanied by a decrease in LVET. The increase in HR after dark chocolate consumption may be attributed to certain stimulants it contains, such as caffeine and theobromine [55]. However, these findings were primarily noted in athletes over 40 years, as indicated by the subgroup analyses. This occurred because middle-aged individuals, who typically exhibit higher blood pressure, experience vasodilation due to reduced peripheral vascular resistance with dark chocolate consumption [17]. In response to this reduction in blood pressure, the body acts compensatory by increasing cardiac output to ensure adequate blood flow [56].

In addition to the statistical significance of the differences, this study also evaluated the impact of dark chocolate consumption on clinical practice in endurance runners. The resulting values showed that the MID was slightly lower than that reported in the literature. Specifically, studies indicate that for blood pressure the MID can be 2 mmHg, for PWV 1 m/s, and for VO_2_max 1.8 mL/kg/min [57,58,59]. These values are consistent with the findings of the present study. This can be attributed to the fact that MID often varies depending on the domain, the population group, and the intervention. Most studies assess the MID in patients and the general population. Endurance runners represent a unique population with specific exercise-induced physiological adaptations. Thus, our results indicated that even the smallest changes observed from the consumption of dark chocolate had significant practical value for endurance runners, as even minor improvements made a noticeable difference in performance. The proportion of 87% of the participants who reported improvement in their athletic performance reinforces the clinical significance of the calculated MID. This indicates that the statistically significant changes observed are not only statistically significant but also clinically significant for the athletes, suggesting that the MID derived from the statistical analysis is representative of the actual improvement in their athletic performance.

The improvement in endurance runners’ arterial stiffness markers, such as PWV and AIx, has significant practical implications for their performance and long-term health [49]. With more elastic arteries, runners can better handle pressure fluctuations during exercise, reducing the risk of cardiovascular problems [60]. This is particularly important for the long-term health of runners [61]. The improved muscle blood flow during exercise can enhance performance, especially in long-distance events [17]. With vascular dilation and reduced PWV and AIx, blood pressure is also lowered, making the heart’s workload easier [17,62]. This means that runners’ hearts need to work less hard to maintain blood flow during exercise, reducing fatigue and improving endurance. Improved blood circulation and reduced cardiac load may contribute to faster recovery after intense exercise [63]. This knowledge empowers runners to make informed decisions about their training and dietary habits, potentially enhancing their performance and health.

Notable findings also came from the correlation analysis conducted. Specifically, PWV, as the primary predictive marker, was found to be positively associated with age, BMI, blood pressure, and resting heart rate, with significant correlations seen in both the initial and final measurements. However, there was a lack of correlation between these factors and PWV variability, i.e., the difference between the initial and final measurements. This finding indicates that the intervention with dark chocolate consumption improved arterial stiffness independently of the influence of the above factors, thereby confirming its beneficial effect and reassuring the audience about the robustness of the study’s conclusions.

### Limitations

The study’s main limitations were the sex of the participants, as female runners were not included, and the absence of a control or placebo group, such as sedentary individuals, power-trained athletes, or those consuming white chocolate. This exclusion eliminated the potential impact of hormonal changes that women experience during menstruation, perimenopause, and menopause, which are known to affect vascular function. Additionally, there was no follow-up after the intervention to examine whether and when the participants returned to their baseline condition, as the study focused solely on the acute effects of dark chocolate consumption. It is possible that the observed effect might not be solely related to the polyphenols in dark chocolate, but rather to the additional calories consumed during recovery between training sessions. The term ‘recovery’ refers to the period following exercise when athletes replenish their energy stores, particularly glycogen, and allow muscle repair. During this time, additional calories are consumed to support the recovery process. While the polyphenols in dark chocolate are believed to play a key role in the observed effects, the contribution of the extra calories consumed during this recovery period might also influence the outcomes. The extra energy intake from the chocolate could have contributed to improved recovery and performance and might have outweighed the bioactive effect of the polyphenols in dark chocolate. Furthermore, the potential for unknown confounders, multiple testing, and the risk of Type I and Type II errors, especially in subgroup analyses due to the sample size, should be considered. The study only examines short-term effects over two weeks. The long-term benefits and potential side effects of regular dark chocolate consumption would be important to explore.

## 5. Conclusions

In conclusion, this study demonstrated that 2 weeks of daily dark chocolate consumption significantly reduces the strongest predictive markers of arterial stiffness in male endurance runners, thereby improving their arterial function. This finding strengthens the well-established favorable effect of aerobic exercise on arterial health. The combination of endurance training and dark chocolate intake may have a long-term positive impact on vascular elasticity and overall cardiovascular health. However, further research is needed to confirm these effects in larger, placebo-controlled, and more diverse populations, including women and individuals following different training regimens.

While flavanols in dark chocolate offer significant health benefits, it is important to recognize that chocolate is a calorie-dense product. Maintaining a balanced caloric intake is essential to avoid unintended weight gain or obesity. Therefore, enjoying chocolate in moderation can be a delightful and health-conscious choice, contributing to both dietary satisfaction and overall well-being.

Future research should focus on identifying which specific compounds in dark chocolate contribute to its beneficial effects on arterial function. Key areas for investigation include flavonoids, catechins, theobromine, and other phytochemicals, evaluating their individual and combined effects on arterial health. It is also crucial to explore how factors like cocoa content, added sugars, and the presence of ingredients such as nuts or dairy affect cardiovascular outcomes. Longitudinal studies assessing the long-term benefits of dark chocolate consumption in controlled dietary contexts could offer deeper insights into its lasting health impacts.

## Figures and Tables

**Table 1:** Chemical analysis of chocolate.

Component	Polyphenols (as Tyrosol) (mg/Kg)	Catechins (Catechin-Epicatechin) (mg/Kg)
Content	24,450	11,550
Method	COI/T.20/Doc No 29	HPLC-UV
Method Code	M130/GF	M129/GF
Method Reference Limit	40	1

**Table 2:** Clinical characteristics of the participants.

	Mean ± SD (n = 46)	Mean ± SD (n = 23) <40 yrs	Mean ± SD (n = 23) >40 yrs
Age (years)	40.69 ± 9.04	33.13 ± 4.89	48.26 ± 4.85
Weight (kg)	73.69 ± 9.40	72.48 ± 10.04	74.91 ± 8.77
Height (cm)	175.86 ± 7.61	176.65 ± 7.63	175.09 ± 7.69
ΒΜΙ (kg/m^2^)	23.79 ± 2.35	23.17 ± 2.31	24.42 ± 2.48
HR (bpm)	57.34 ± 6.70	54.43 ± 7.19	56.26 ± 6.18
SBP (mmHg)	128.58 ± 8.40	124.61 ± 8.94	126.57 ± 7.90
DBP (mmHg)	76.47 ± 6.78	73.96 ± 6.53	75.00 ± 7.12
T-E (yrs)	9.98 ± 6.03	10.43 ± 6.69	9.52 ± 5.40

Mean: average value; SD: standard deviation; BMI: body mass index; HR: heart rate; SBP: Systolic Blood Pressure; DBP: Diastolic Blood Pressure; T-E: training experience.

**Table 3:** Results of initial and final measurement of arterial function indices—age 25–55 years.

		Baseline	Follow-Up	Difference				
		Mean ± SD (n = 46)	Mean ± SD (n = 46)	Mean ± SD	*t*	*p*	Cohen’s d	MID
HR (rest) (bpm)		57.59 ± 8.82	60.22 ± 10.11	−2.63 ± 8.03	−2.221	0.031 *	−0.327	1.606
bSBP (mmHg)		128.39 ± 11.59	125.67 ± 11.52	2.72 ± 7.39	2.494	0.016 *	0.368	1.478
bDBP (mmHg)		76.67 ± 9.49	74.54 ± 9.11	2.13 ± 6.33	2.284	0.027 *	0.337	1.266
bPP (mmHg)		51.72 ± 10.61	51.13 ± 8.61	0.59 ± 7.72	0.516	0.609	0.076	1.544
bMAP (mmHg)		93.74 ± 8.99	91.48 ± 9.06	2.26 ± 5.65	2.714	0.009 *	0.400	2.825
cSBP (mmHg)		121.50 ± 13.94	120.00 ± 12.72	1.50 ± 10.67	0.954	0.345	0.141	2.134
cDBP (mmHg)		76.38 ± 9.18	74.24 ± 9.01	2.14 ± 6.32	2.264	0.029 *	0.339	1.264
cPP (mmHg)		44.83 ± 12.18	45.46 ± 12.07	−0.63 ± 9.22	−0.464	0.645	−0.068	1.844
cMAP (mmHg)		93.45 ± 8.68	91.18 ± 9.02	2.27 ± 5.55	2.614	0.009 *	0.404	2.775
cf-PWV (m/s)		7.78 ± 1.24	6.86 ± 1.20	0.92 ± 0.94	6.606	0.000 **	0.974	0.752
AIx (%)		5.56 ± 14.80	−13.91 ± 22.67	19.47 ± 22.87	5.775	0.000 **	0.852	18.296
PPratio		1.18 ± 0.15	1.16 ± 0.17	0.03 ± 0.19	0.915	0.365	0.135	0.038
LVET (msec)		325.83 ± 34.92	309.89 ± 28.69	15.94 ± 31.68	3.412	0.001 **	0.194	6.336
Total Time (mins)		13.46 ± 1.93	13.75 ± 1.98	0.29 ± 0.49	−0.400	0.001 **	−0.590	0.245
HR (max) (bpm)		180.83 ± 9.97	182.91 ± 9.29	−2.09 ± 4.94	−2.863	0.006 *	−0.422	0.988
AT-Time (mins)		9.69 ± 1.49	10.36 ± 1.58	0.67 ± 0.88	−5.209	0.001 *	−0.768	0.440
AT-VO_2_max (%)		78.59 ± 5.97	84.04 ± 5.82	−5.46 ± 6.53	−5.667	0.001 *	−0.835	3.265
VE (L/min)		119.62 ± 17.37	124.12 ± 16.28	−4.50 ± 13.16	−2.316	0.025 *	−0.336	2.632
VO_2_max (ml/kg/min)		52.22 ± 8.70	53.43 ± 8.44	−1.21 ± 3.61	−2.274	0.028 *	−0.335	0.722
VO_2_max/Pred (%)		133.96 ± 24.86	136.57 ± 22.92	−2.61 ± 9.54	−1.855	0.070	−0.274	1.908

Mean: average value; SD: standard deviation; MID: minimal important difference; the bold is statistically significant *p*-values, *: *p* < 0.05; **: *p* < 0.001. HR: heart rate; bSBP: brachial; bDBP: brachial Diastolic Blood Pressure; bPP: brachial pulse pressure; bMAP: brachial Mean Arterial Pressure; cSBP: central Systolic Blood Pressure; cDBP: central Diastolic Blood Pressure; cPP: central pulse pressure; cMAP: central Mean Arterial Pressure; cf-PWV: carotid–femoral pulse wave velocity; AIx: augmentation index; PPratio: pulse pressure ratio; LVET: left ventricular ejection time; AT: anaerobic threshold; VE: ventilation.

**Table 4:** Results of initial and final measurement of arterial function indices—age < 40 years.

		Baseline	Follow-Up	Difference				
		Mean ± SD (n = 23)	Mean ± SD (n = 23)	Mean ± SD	*t*	*p*	Cohen’s d	MID
HR (rest) (bpm)		57.22 ± 10.48	58.35 ± 8.91	−1.13 ± 7.54	−0.719	0.480	−0.150	1.508
bSBP (mmHg)		127.43 ± 12.14	124.74 ± 10.87	2.70 ± 6.92	1.867	0.075	0.389	1.384
bDBP (mmHg)		73.57 ± 7.95	71.70 ± 8.29	1.87 ± 7.05	1.271	0.217	0.265	1.410
bPP (mmHg)		53.87 ± 12.55	53.04 ± 8.91	0.83 ± 7.63	0.519	0.609	0.108	1.526
bMAP (mmHg)		91.35 ± 7.49	89.22 ± 8.12	2.13 ± 6.06	1.685	0.106	0.351	1.212
cSBP (mmHg)		120.91 ± 13.67	121.48 ± 13.37	−0.57 ± 12.27	−0.221	0.827	−0.046	2.454
cDBP (mmHg)		73.28 ± 7.66	71.41 ± 8.09	1.87 ± 7.03	1.271	0.217	0.265	1.406
cPP (mmHg)		47.35 ± 14.33	49.78 ± 13.88	−2.44 ± 10.79	−1.082	0.291	−0.226	2.158
cMAP (mmHg)		91.06 ± 7.20	89.02 ± 7.92	2.04 ± 6.03	1.685	0.106	0.351	1.206
cf-PWV (m/s)		7.47 ± 1.20	6.57 ± 1.19	0.90 ± 0.62	6.898	0.000 **	1.438	0.496
AIx (%)		2.18 ± 15.35	−24.11 ± 25.42	26.30 ± 27.46	4.593	0.000 **	0.958	21.968
PPratio		1.17 ± 0.14	1.10 ± 0.19	0.07 ± 0.22	1.533	0.140	0.320	0.044
LVET (msec)		326.48 ± 40.21	315.43 ± 32.11	11.04 ± 25.77	2.055	0.052	0.429	12.885
Total Time (mins)		13.91 ± 2.19	14.22 ± 2.26	0.31 ± 0.47	−3.207	0.004 *	−0.669	0.235
HR (max) (bpm)		183.43 ± 9.25	185.26 ± 8.94	−1.83 ± 4.89	−1.792	0.087	−0.374	0.978
AT-Time (mins)		9.90 ± 1.75	10.65 ± 1.84	0.75 ± 1.06	−3.383	0.003 *	−0.705	0.530
AT-VO_2_max (%)		78.30 ± 6.60	83.26 ± 6.68	−4.96 ± 7.54	−3.155	0.005 *	−0.658	3.770
VE (L/min)		120.24 ± 18.91	127.61 ± 18.71	−7.37 ± 13.74	−2.572	0.017 *	−0.536	6.870
VO_2_max (ml/kg/min)		53.22 ± 10.21	54.82 ± 9.43	−1.60 ± 3.71	−2.075	0.050	−0.433	1.484
VO_2_max/Pred (%)		124.30 ± 23.19	127.91 ± 20.89	−3.61 ± 8.79	−1.969	0.062	−0.411	3.516

Mean: average value; SD: standard deviation; MID: minimal important difference; the bold is statistically significant *p*-values, *: *p* < 0.05; **: *p* < 0.001. HR: heart rate; bSBP: brachial; bDBP: brachial Diastolic Blood Pressure; bPP: brachial pulse pressure; bMAP: brachial Mean Arterial Pressure; cSBP: central Systolic Blood Pressure; cDBP: central Diastolic Blood Pressure; cPP: central pulse pressure; cMAP: central Mean Arterial Pressure; cf-PWV: carotid–femoral pulse wave velocity; AIx: augmentation index; PPratio: pulse pressure ratio; LVET: left ventricular ejection time; AT: anaerobic threshold; VE: ventilation.

**Table 5:** Results of initial and final measurement of arterial function indices—age > 40 years.

		Baseline	Follow-Up	Difference				
		Mean ± SD (n = 23)	Mean ± SD (n = 23)	Mean ± SD	*t*	*p*	Cohen’s d	MID
HR (rest) (bpm)		57.96 ± 6.99	62.52 ± 10.20	−4.57 ± 8.27	−2.648	0.015 *	−0.552	4.135
bSBP (mmHg)		129.35 ± 11.20	126.61 ± 12.30	2.74 ± 7.98	1.645	0.114	0.343	1.596
bDBP (mmHg)		79.78 ± 10.04	77.39 ± 9.17	2.39 ± 5.66	2.028	0.055	0.423	2.830
bPP (mmHg)		49.57 ± 7.93	49.22 ± 8.05	0.348 ± 7.98	0.209	0.836	0.044	1.596
bMAP (mmHg)		96.13 ± 9.86	93.74 ± 9.55	2.39 ± 5.34	2.147	0.043 *	0.448	2.670
cSBP (mmHg)		122.09 ± 14.50	118.52 ± 12.17	3.57 ± 8.56	1.996	0.058	0.416	4.280
cDBP (mmHg)		79.49 ± 9.75	77.10 ± 8.88	2.39 ± 5.37	2.008	0.051	0.423	2.685
cPP (mmHg)		42.30 ± 9.20	41.13 ± 8.14	1.17 ± 7.11	0.791	0.437	0.165	1.422
cMAP (mmHg)		95.84 ± 9.57	93.45 ± 9.26	2.39 ± 5.15	2.118	0.041 *	0.448	2.575
cf-PWV (m/s)		8.08 ± 1.22	7.14 ± 1.16	0.94 ± 1.19	3.771	0.001 *	0.786	0.595
AIx (%)		8.94 ± 13.74	−3.71 ± 13.72	12.65 ± 14.78	4.106	0.000 **	0.856	11.824
PPratio		1.20 ± 0.16	1.21 ± 0.14	−0.02 ± 0.16	−0.487	0.631	−0.102	0.032
LVET (msec)		325.17 ± 29.62	304.35 ± 24.26	20.83 ± 36.59	2.730	0.012 *	0.569	18.295
Total Time (mins)		13.00 ± 1.54	13.27 ± 1.56	0.27 ± 0.52	−2.443	0.023 *	−0.509	0.260
HR (max) (bpm)		178.22 ± 10.23	180.57 ± 9.23	−2.35 ± 5.09	−2.210	0.038 *	−0.461	2.545
AT-Time (mins)		9.47 ± 1.16	10.07 ± 1.25	0.60 ± 0.66	−4.358	0.000 **	−0.909	0.528
AT-VO_2_max (%)		78.87 ± 5.41	84.83 ± 4.82	−5.96 ± 5.47	−5.220	0.000 **	−1.088	4.376
VE (L/min)		119.00 ± 16.10	120.62 ± 12.91	−1.62 ± 12.18	−0.639	0.530	−0.133	2.436
VO_2_max (ml/kg/min)		51.40 ± 7.15	52.04 ± 7.27	−0.64 ± 3.70	−0.833	0.414	−0.174	0.740
VO_2_max/Pred (%)		143.61 ± 23.06	145.22 ± 21.94	−1.61 ± 10.33	−0.747	0.463	−0.156	2.066

Mean: average value; SD: standard deviation; MID: minimal important difference; the bold is statistically significant p-values, *: *p* < 0.05; **: *p* < 0.001. HR: heart rate; bSBP: brachial; bDBP: brachial Diastolic Blood Pressure; bPP: brachial pulse pressure; bMAP: brachial Mean Arterial Pressure; cSBP: central Systolic Blood Pressure; cDBP: central Diastolic Blood Pressure; cPP: central pulse pressure; cMAP: central Mean Arterial Pressure; cf-PWV: carotid–femoral pulse wave velocity; AIx: augmentation index; PPratio: pulse pressure ratio; LVET: left ventricular ejection time; AT: anaerobic threshold; VE: ventilation.

**Table 6:** Assessment of the participants’ opinions on the effect of dark chocolate consumption based on the Likert scale.

How Would You Rate the Effects of Dark Chocolate Consumption?		
Rating	Description	Percentage (%)
1	Very positive	60
2	Positive	27
3	Similar	10
4	Negative	3
5	Very negative	0

## Data Availability

Due to ethical and privacy considerations, the data supporting the findings of this study are not publicly available.
